# Genetic Evolution of *Mycobacterium abscessus* Conferring Clarithromycin Resistance during Long-Term Antibiotic Therapy

**DOI:** 10.1155/2020/7623828

**Published:** 2020-03-28

**Authors:** Bing Li, Qi Guo, Yanhua Mao, Yuzhen Zou, Yongjie Zhang, Zhemin Zhang, Haiqing Chu

**Affiliations:** ^1^Department of Respiratory Medicine, Shanghai Pulmonary Hospital, Tongji University School of Medicine, Shanghai 200433, China; ^2^Tongji University School of Medicine, Shanghai 200092, China; ^3^Shanghai Key Laboratory of Tuberculosis, Shanghai Pulmonary Hospital, Tongji University School of Medicine, Shanghai 200433, China

## Abstract

**Objectives:**

Clarithromycin is recommended as the core agent for treating *M. abscessus* infections, which usually calls for at least one year of treatment course, facilitating the development of resistance. This study aimed to identify the underlying mechanism of *in vivo* development of clarithromycin resistance in *M. abscessus* clinical isolates.

**Methods:**

*M. abscessus* isolates from patients with lung infections during long-term antibiotic therapy were longitudinally collected and sequenced. PFGE DNA fingerprinting was used to confirm the genetic relationships of the isolates. Whole genome comparative analysis was performed to identify the genetic determinants that confer the clarithromycin resistance.

**Results:**

Three pairs of initially clarithromycin-susceptible and subsequently clarithromycin-resistant *M. abscessus* isolates were obtained. We found that the clarithromycin-resistant isolates emerged relatively rapidly, after 4–16 months of antibiotic therapy. PFGE DNA fingerprinting showed that the clarithromycin-resistant isolates were identical to the initial clarithromycin-susceptible ones. Whole genome sequencing and bioinformatics analysis identified several genetic alternations in clarithromycin-resistant isolates, including genes encoding efflux pump/transporter, integral component of membrane, and the *tetR* and *lysR* family transcriptional regulators.

**Conclusion:**

We identified genes likely encoding new factors contributing to clarithromycin-resistance phenotype of *M. abscessus*, which can be useful in prediction of clarithromycin resistance in *M. abscessus*.

## 1. Introduction

Nontuberculous mycobacteria (NTM) refer to mycobacterial species other than those of *Mycobacterium tuberculosis* complex and *Mycobacterium leprae* [1]. It has been increasingly recognized that NTM are important human pathogens causing distinct infections with clinical manifestations similar to tuberculosis, such as chronic pulmonary disease, cutaneous disease, lymphadenitis, and disseminated disease [1]. Among them, chronic pulmonary disease is the most frequently encountered syndrome in clinical practice [2]. *Mycobacterium abscessus* has been reported as the second most common pathogen, after *Mycobacterium avium* complex, in NTM lung disease, and is increasing in incidence [3]. Pulmonary disease caused by *M. abscessus* is associated with substantial morbidity and mortality [4].

As *M. abscessus* is intrinsically resistant to multiple antibiotic classes, treatment of infections caused by *M. abscessus* is infamously difficult [5]. Until now, no reliable antibiotic regimen has been established for *M. abscessus* pulmonary disease. Current guidelines recommend an oral macrolide-based (mainly clarithromycin) antibiotic therapy, combined with intravenous amikacin with cefoxitin or imipenem, based on the results of drug susceptibility testing [1]. However, the cure rate for *M. abscessus* lung disease is low (about 30–50%), and the recurrence rate is high, even after successful treatment completion [6, 7].

The poor treatment outcomes of *M. abscessus* lung diseases are attributable to the resistance to clarithromycin of *M. abscessus*, including acquired resistance (resistance at day 3) and inducible resistance (susceptible at day 3 but resistant at day 14). The mainly reported mechanism underlying clarithromycin resistance involved alternation in the drug target sites, including mutations in the 23S rRNA gene (*rrl*) which confer acquired resistance [8] and modifications in the rRNA by a functional methyltransferase *erm* (41) gene that confer inducible resistance [9]. However, most data presented before were from single time point studies during patient treatment. As the treatment course on *M. abscessus* lung diseases calls for continuation of antibiotic therapy lasting at least 12 months, clarithromycin resistance can develop during the long treatment period [10]. Understanding the mechanisms underlying the *de novo* development of clarithromycin resistance during treatment is important for optimizing treatment of infection while avoiding the emergence of resistance. In this regard, longitudinal monitoring of clarithromycin susceptibility before and throughout antibiotics therapy is critical.

In this study, isogenic paired isolates of *M. abscessus* demonstrating initially susceptible and subsequently resistant during antibiotic therapy to clarithromycin were obtained from the same patients, which enabled us to compare the susceptible and resistant strains and explore the underlying molecular mechanisms of *in vivo* development of acquired resistance to clarithromycin. We found that the acquired resistant isolates against clarithromycin emerged relatively rapidly, after 4–16 months of antibiotic therapy. By bioinformatic analyses of the whole genomes of the serial *M. abscessus* isolates, several important genetic variants were identified in the subsequently resistant isolates, including genes encoding efflux pump/transporter, integral component of membrane, and the *tetR* and *lysR* family transcriptional regulators, which have been proposed as antibiotic resistance-associated factors in other bacteria. This work provides insight into how *M. abscessus* develops resistance *in vivo* in response to the selective pressure of antibiotic treatment.

## 2. Materials and Methods

### 2.1. *M. abscessus* Isolates and Clinical Data Collection

During January 2014 to December 2017, three paired clinical isolates of *M. abscessus*, which were initially clarithromycin susceptible and then became clarithromycin resistant, were collected from sputum of patients with lung infections at Shanghai Pulmonary Hospital. Clinical data from patients were collected by chart review.

### 2.2. Bacterial Identification and Antimicrobial Susceptibility Testing

Species of isolates were preliminarily screened for NTM by both the MGIT960 medium culture and p-nitrobenzoic acid test, followed by molecular identification of *M. abscessus* by sequencing the *rpoB* and *erm* (41) genes and were finally confirmed by whole genome sequencing. All isolates were then stored at −80°C until use. Antimicrobial susceptibility for clarithromycin was determined by the broth microdilution method according to CLSI guidelines and was repeated in triplicate. The breakpoints were interpreted according to (CLSI)-M24-A2 as follows: susceptible, minimal inhibitory concentration (MIC) ≤ 2 mg/L; intermediate, MIC = 4 mg/L; resistant, MIC ≥ 8 mg/L. Antimicrobial susceptibility for other antibiotics, including amikacin, sulfonamides, moxifloxacin, cefoxitin, doxycycline, tigecycline, linezolid, imipenem, and tobramycin, was determined by the Sensititre RAPMYCO panel. *Mycobacterium peregrinum* (ATCC 700686; American Type Culture Collection, Manassas, VA, USA) and *Staphylococcus aureus* (ATCC 29213; American Type Culture Collection, Manassas, VA, USA) served as the control reference strains.

### 2.3. Phylogenetic Distance Analysis Using Pulsed-Field Gel Electrophoresis (PFGE)

The phylogenetic distance among *M. abscessus* isolates was analyzed by PFGE with modifications of the methods as described previously [11]. In brief, *M. abscessus* isolates were cultured to the logarithmic phase at 37°C in the 7H9 medium and were collected and resuspended in cell suspension buffer (CSB) to an OD600 of 1.6. Cell suspensions were mixed in a 1 : 1 ratio with 1% SeaKem Gold agarose (containing 1% SDS) and pipetted into disposable plug moulds (Bio-Rad). The plugs were lysed with 20 mg/ml lysozyme at 37°C for 3 h. The lysis solution was replaced with cell lysis buffer (CLB) [50 mM Tris/HCl (pH 8.0), 50 mM EDTA, and 0.1 mg/ml proteinase K] and incubated at 56°C overnight with shaking (160 rounds per minute). The plugs were washed once with ddH2O in a shaking water bath at 56°C for 15 min and then five times with Tris (10 mM)/EDTA (1 mM) buffer (pH 8.0) for 15 min each.

The plugs containing the genomic DNA were digested with DraI for 4 h at 37°C. Digested DNA was then separated on a 1% agarose gel using a CHEF-DRIII system (Bio-Rad Laboratories, Hercules, CA) in 0.5°×°TBE buffer [1 M Tris/HCl (pH 8.0), 0.9 M boric acid, and 10 mM Na_2_EDTA] with 200 *μ*M thiourea. Pulse time was ramped from 5 to 35 s for 19 h at 14°C and a constant voltage of 6 V/cm. Similarity between PFGE patterns was analyzed by computerized band analysis with Bionumerics software version 3.3.

### 2.4. Detection of Mutations in *rrl* and *erm* (41)

The whole genomes of the 6 *M. abscessus* isolates in this study have been previously sequenced by us (NCBI bioproject PRJNA488058) [12]. Sequences of *rrl* and *erm* (41) were extracted from the sequencing data. The presence of mutations in the *rrl* and *erm* (41) genes was detected by sequence alignment with the homologous sequences of the reference mycobacterial strains *M. abscessus* AATCC 19977 by BLAST.

### 2.5. Bioinformatic Analyses of the Whole Genome Sequencing Data

Sequencing reads were initially mapped to the reference genome *M. abscessus* ATCC19977 (GenBank accession no. NC_010397.1) using Snippy v4.3.0/BWA-MEM v0.7.11 (https://github.com/tseemann/snippy). Variants were called using Snippy v4.3.0/Freebayes v1.1.0-60. The unique variants in subsequent acquired resistant isolates against clarithromycin were identified by comparing the detected variants in subsequently clarithromycin-resistant isolates and in initially clarithromycin-susceptible isolates using bcftools v1.7-17.

## 3. Results

### 3.1. Demographic and Clinical Features

**Figure 1:**
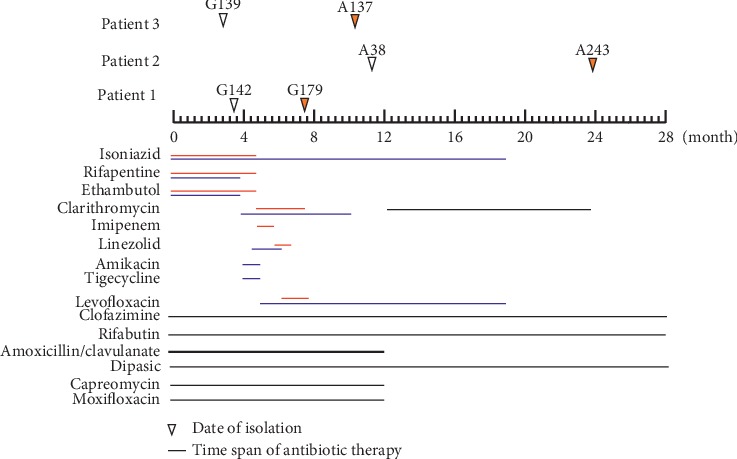
Timeline of antibiotic usage and date of *M. abscessu*s isolation. Triangle filled with white indicates that the strain was susceptible to clarithromycin at day 3, whereas yellow triangle represent clarithromycin resistance at day 3. All of the isolates were isolated from sputum.

*M. abscessus* isolates that were initially clarithromycin susceptible and then acquired clarithromycin resistance from a same patient were isolated from three patients during the study period.  Table 1 shows the demographic and clinical features of the three cases. The BMI of patients was relatively low, implying that patients were underweight. All patients had bronchiectasis. Patient 2 was coinfected with *M. tuberculosis*, leaving the treatment vastly complicated. The most common clinical symptoms were cough and sputum. Computed tomography showed that all patients had bilateral bronchiectasis with multiple cavities, which was in agreement with another report [13]. As the three cases had initial positive AFB smears, all of them received multidrug combinations of antituberculosis treatment before the diagnosis of *M. abscessus* lung disease (the detailed antibiotic usage is shown in Figure 1). Though the patients were given a continuation of long-term multitherapy, their symptoms and radiological results were not improved. Two of them even progressed after the treatment and failed to convert to sputum smear-negative.

### 3.2. Microbiological Characteristics of Clinical Isolates of *M. abscessus*

The characteristics of six *M. abscessus* isolates are shown in Table 2. Isolates were highly resistant to almost all the antibiotics tested, except for amikacin and tigecycline. All strains belonged to genotype A, in which the strains harbored a functional *erm* (41) mediating inducible clarithromycin resistance. As a consequent, all isolates had a high MIC to clarithromycin at day 14, and the MIC in subsequent isolates was higher than in initial isolates. At day 3, the initial strain G142, A38, and G139 were susceptible to clarithromycin. However, after a period of antibiotic treatment, the corresponding subsequent isolates G179, A243, and A137 became resistant to clarithromycin (developed into acquired clarithromycin-resistance phenotype) without any mutation in the drug target gene *rrl*, indicating the presence of other factors contributing to the resistance.

### 3.3. *In Vivo* Evolution of Clarithromycin Resistance in *M. abscessus*

The use of antimicrobial agents throughout the treatment course was analyzed (Figure 1). As patient 1 and patient 3 had a sputum smear-positive but culture-negative results, they received the empirical antituberculosis treatment with isoniazid, rifapentine, and ethambutol for about 4 months prior to the diagnosis of *M. abscessus* lung diseases. Until *M. abscessus* was cultivated from the sputum, treatment regimens were switched to clarithromycin, amikacin, tigecycline, imipenem, linezolid, and levofloxacin, based on the antibiotic susceptibility results. Since patient 2 had a prior drug-resistant tuberculosis and had severe drug-induced liver injury, treatment for him was extremely difficult. He received a total of seven kinds of antibiotics which included the second-line anti-TB drugs and those possessing antimicrobial activity against *M. abscessus*. Under the long-term selective pressure of various antibiotics, acquired resistant isolates of *M. abscessus* against clarithromycin, G179, A243, and A317 emerged after 4–16 months of treatment.

**Figure 2:**
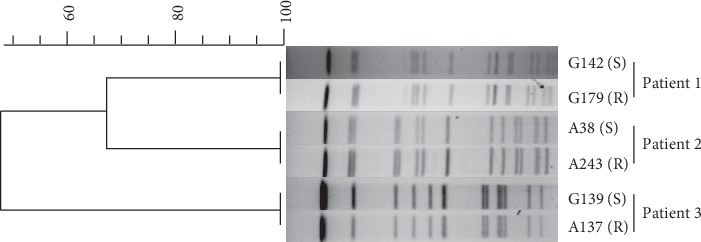
Pulsed-field gel electrophoresis (PFGE) analysis of the phylogenetic distance of *M. abscessus* isolated from the same patient. The dendrogram was constructed using BioNumerics software Version 6.6 (Applied Maths, St-Martens-Latem, Belgium). S represents susceptible to clarithromycin; R represents resistant to clarithromycin.

To assess whether the subsequently clarithromycin-resistant isolates, G179, A243, and A317, were derived from the corresponding initially clarithromycin-susceptible isolates G142, A38, and G139, the phylogenetic distance of these isolates was analyzed. PFGE DNA fingerprinting showed that the clarithromycin-resistant isolate was identical to the initially clarithromycin-susceptible isolate among all the three cases (Figure 2). Thereby, clarithromycin-resistance phenotype in the subsequent strains was considered as the result of genetic variation from the initial strains.

### 3.4. Identification of Genetic Determinants Conferring Clarithromycin Resistance

It has been widely reported that the acquired clarithromycin resistance in *M. abscessus* is due to mutations in the *rrl* gene [14, 15]. However, the resistance phenotype in strains G179, A243, and A317 could not be explained by this mechanism, as no mutation was found in G179 and A243, and only one point mutation of C3042T was observed in A317, which also existed in the susceptible strain G139. These results indicated the presence of other yet-to-be identified mechanisms in these subsequent acquired clarithromycin-resistance strains. To identify the genetic determinants that confer the clarithromycin resistance in these strains, whole genomes of the initially clarithromycin-susceptible isolates were compared with those of the corresponding subsequently clarithromycin-resistant isolates.

**Figure 3:**
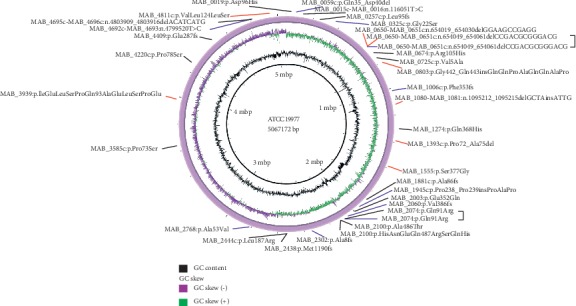
Mutation profiles of clarithromycin-acquired resistant *M. abscessus.* We showed mutations present in 3 clarithromycin-acquired resistant *M. abscessus* strains compared to the initially clarithromycin-susceptible strains. Variants in red indicate that the variants exist in G179 compared with G142; variants in black indicate that the variants exist in A243 compared with A38; variants in blue indicate that the variants exist in A137 compared with G139. ATCC 19977 served as the reference genome.

Mutation profiles are shown in Figure 3. A total of 35 high quality variants, 8 in G179, 17 in A243, and 10 in A317, were identified, which were distributed in 5 intergenic regions and 28 open reading frames (synonymous mutations in the ORF were excluded). Among them, notable mutations were included in several efflux pump/transporter genes (Mab_2302, Mab_1006c), the integral component of membrane (Mab_0803, Mab_1555), the *tetR* family transcriptional regulator (Mab_1881c), and *lysR* family transcriptional regulator (Mab_4409). Other important mutations were distributed in the intergenic regions, including the interval of porins MspA-MspA (Mab_1080-Mab_1081) and the upstream of ESX-1 secretion-associated regulator EspR (Mab_0115c). Interestingly, two variants (Mab_2074, Mab_0650-Mab_0651) were simultaneously identified in two subsequent isolates acquiring clarithromycin resistance.

## 4. Discussion

In this study, three paired isogenic isolates of *M. abscessus* were longitudinally collected from three patients with lung infections during the course of antibiotic therapy, allowing us to monitor and study the development of clarithromycin resistance *in vivo*. Clarithromycin-acquired resistance emerged relatively rapidly, after 4–16 months of multidrug antibiotic treatment. This finding highlights the importance of rational use of antibiotics in clinical practice as well as monitoring clarithromycin susceptibility of the pathogen during the course of therapy. Different from previously reported acquired clarithromycin-resistant isolates of *M. abscessus* in which *rrl* was the mutant and resulted in altered drug target site, we uncovered several other important variants in the subsequently clarithromycin-acquired resistant isolates, potentially representing new factors contributing to the acquired clarithromycin-resistance phenotype of *M. abscessus.*

NTM lung infection is difficult to diagnose due to the nonspecific symptoms which are similar to the those of pulmonary TB [16, 17]. Therefore, additional microbiologic diagnostics are essential. Smear microscopy and mycobacterial culture are commonly used for identification of mycobacteria. However, smear microscopy cannot differentiate NTM from *M. tuberculosis*, and the culture requires several weeks before results can be obtained [18, 19]. As such, patients with positive smears will be empirically prescribed antituberculosis therapy. In this study, patient 1 and patient 3 received isoniazid, rifapentine, and ethambutol for about 4 months prior to the diagnosis of *M. abscessus* lung disease. No improvements in symptoms and radiological results were observed throughout the treatment course, suggesting that further investigations are desperately needed for the development of methods that could rapidly detect and identify mycobacteria into precise species.

The treatment of *M. abscessus* lung disease is more difficult than that caused by other NTM, owing to the bacterium's high-level resistance [20, 21]. The efficacy and safety of the current guideline lack data support. In our study, treatment regimens in patient 1 and patient 3 were switched to anti-*M. abscessus* antibiotics according the guideline and susceptibility test results after the diagnosis of *M. abscessus* lung disease. However, because of adverse drug reactions, inconvenience to patients, and high costs, both patients did not complete the treatment course of imipenem, amikacin, tigecycline, and linezolid. Patient 2 was coinfected with drug-resistant *M. tuberculosis* and had drug-induced liver injury. He was treated with second-line anti-TB agents combined with drugs processing antimicrobial activity against *M. abscessus* [22]. Though relative standard regimens were applied for these patients, the clinical outcomes were discouraging. There is a dearth of research, including animal studies or clinical trials, exploring novel treatment regimens to improve clinical outcomes of *M. abscessus* infections.

It has been reported that during the long-term use of macrolides, *M. abscessus* strains with acquired clarithromycin resistance have emerged due to mutations in the corresponding *rrl* genes [10]. In this study, none of the subsequent clarithromycin-acquired resistant isolates had mutations in the *rrl*, suggesting a new mechanism involved in the clarithromycin resistance. By whole genome sequencing and comparative analysis, we identified several genetic determinants that might contribute to the resistance. Among them, two frameshift mutations were found in Mab_1006c and *mmpS* (Mab_2302). Mab_1006c encodes for the MCE (mammalian cell entry) family protein, and many evidences demonstrated that it is a component of lipid ABC transporters. It has been reported that *M. tuberculosis* strains reduce its virulence when becoming more drug resistant [23, 24]. The mutation of MCE family protein might be the result of selective pressure such as antibiotic therapy. MmpS/MmpL is an efflux pump in *Mycobacterium* species, and mutation in *mmpS* was reported responsible for clarithromycin resistance in strain A317. TetR is a large family of transcriptional regulators. The most frequently characterized function of TetR proteins is regulation of efflux pumps and transporters. Richard et al. showed that mutations in the TetR family transcriptional regulator (MAB_4384) of *M. abscessus* led to upregulation of the MmpS5/MmpL5 efflux pump, thereby increasing the MIC to thiacetazone derivatives [25]. In our study, the frameshift mutation in Mab_1881c (the TetR family transcriptional regulator) was loss-of-function mutation, which might contribute to the clarithromycin resistance in strain A243.

One limitation of our study is that the roles of the variant genes in clarithromycin-acquired resistance have not been tested genetically. We are currently in the process of performing genetics-based tasks to elucidate the functions of these genes in clarithromycin resistance.

## Figures and Tables

**Table 1:** Demographic and clinical features of patients in this study.

Characteristics	Patient 1	Patient 2	Patient 3
Gender	Female	Male	Male
Age (years)	72	49	66
BMI (kg/m^2^)	13.4	17.2	21.4
Underlying diseases	Hypertension; bronchiectasis	Tuberculosis; bronchiectasis; drug-induced liver injury	Hypertension; bronchiectasis; diabetes mellitus
Symptoms	Cough; sputum	Cough; sputum; emaciation	Cough; sputum; chest tightness; hemoptysis
Radiographic characteristics	Bilateral involvement; bronchiectasis, multiple thin wall cavities	Bilateral involvement; bronchiectasis; multiple cavities	Bilateral involvement; bronchiectasis; multiple cavities; pleural effusion
Initial AFB positive	Yes	Yes	Yes
Antibiotics used^a^	H, Rft, E, Clr, Imi, Lzd, Lfx	Cfz, Rfb, Au, Pa, Cm, M, Clr	H, Rft, E, Clr, Lzd, Ami, Tgc, Lfx
Duration of treatment	7 months	28 months	19 months
Radiological result	No change	Progressed	Progressed
Sputum conversion	Withdrawn	Persistent positive sputum culture with TB and *M. abscessus*	Relapse after conversion to negative

^a^H, isoniazid; Rft, rifapentine; E, ethambutol; Clr, clarithromycin; Lzd, linezolid; Imi, imipenem; Lfx, levofloxacin; Ami, amikacin; Tgc, tigecycline; Rfb, rifabutin; Cfz, clofazimine; Pa, dipasic; M, moxifloxacin; Cm, capreomycin; Au, amoxicillin/clavulanate.

**Table 2:** Microbiological characteristics of 6 paired *M. abscessus* isolates.

Variable	Patient 1		Patient 2		Patient 3	
	G142	G179	A38	A243	G139	A317
AMI (amikacin)	>64 (R)	>64 (R)	2 (S)	8 (S)	4 (S)	8 (S)
SXT (sulfonamides)	>8 (R)	>8 (R)	>8 (R)	>8 (R)	>8 (R)	>8 (R)
MXF (moxifloxacin)	8 (R)	8 (R)	>8 (R)	>8 (R)	8 (R)	8 (R)
FOX (cefoxitin)	64 (I)	128 (R)	64 (I)	128 (R)	128 (R)	>128 (R)
DOX (doxycycline)	>16 (R)	>16 (R)	>16 (R)	>16 (R)	8 (R)	16 (R)
TGC (tigecycline)	0.12 (S)	0.12 (S)	0.12 (S)	0.5 (S)	0.5 (S)	4 (R)
LZD (linezolid)	4 (S)	8 (S)	16 (I)	32 (R)	16 (I)	32 (R)
IMI (imipenem)	16 (I)	16 (I)	32 (R)	64 (R)	8 (I)	64 (R)
TOB (tobramycin)	>16 (R)	>16 (R)	4 (I)	8 (R)	4 (I)	>16 (R)
CLA (clarithromycin, D3)	2 (S)	>64 (R)	2 (S)	64 (R)	0.06 (S)	16 (R)
CLA (clarithromycin, D14)	32 (R)	>64 (R)	16 (R)	>64 (R)	16 (R)	>64 (R)
Genotype	A	A	A	A	C3042T	C3042T
*rrl* mutation	None	None	None	None	None	None
Morphotype	Rough	Rough	Rough	Rough	Rough	Rough

S: susceptible; I: intermediate; R: resistant; D3, MIC at day 3; D14, MIC at day 14.

## Data Availability

The data used to support the findings of this study are included within the article.
